# Supplementary material for: Exposure to the RXR Agonist SR11237 in Early Life Causes Disturbed Skeletal Morphogenesis in a Rat Model
Source: Int J Mol Sci. 2019 Oct 20;20(20):5198. doi: 10.3390/ijms20205198 (PMC6829207; doi:10.3390/ijms20205198)
Supplement: Supplementary file 1 [file ijms-20-05198-s001.pdf]

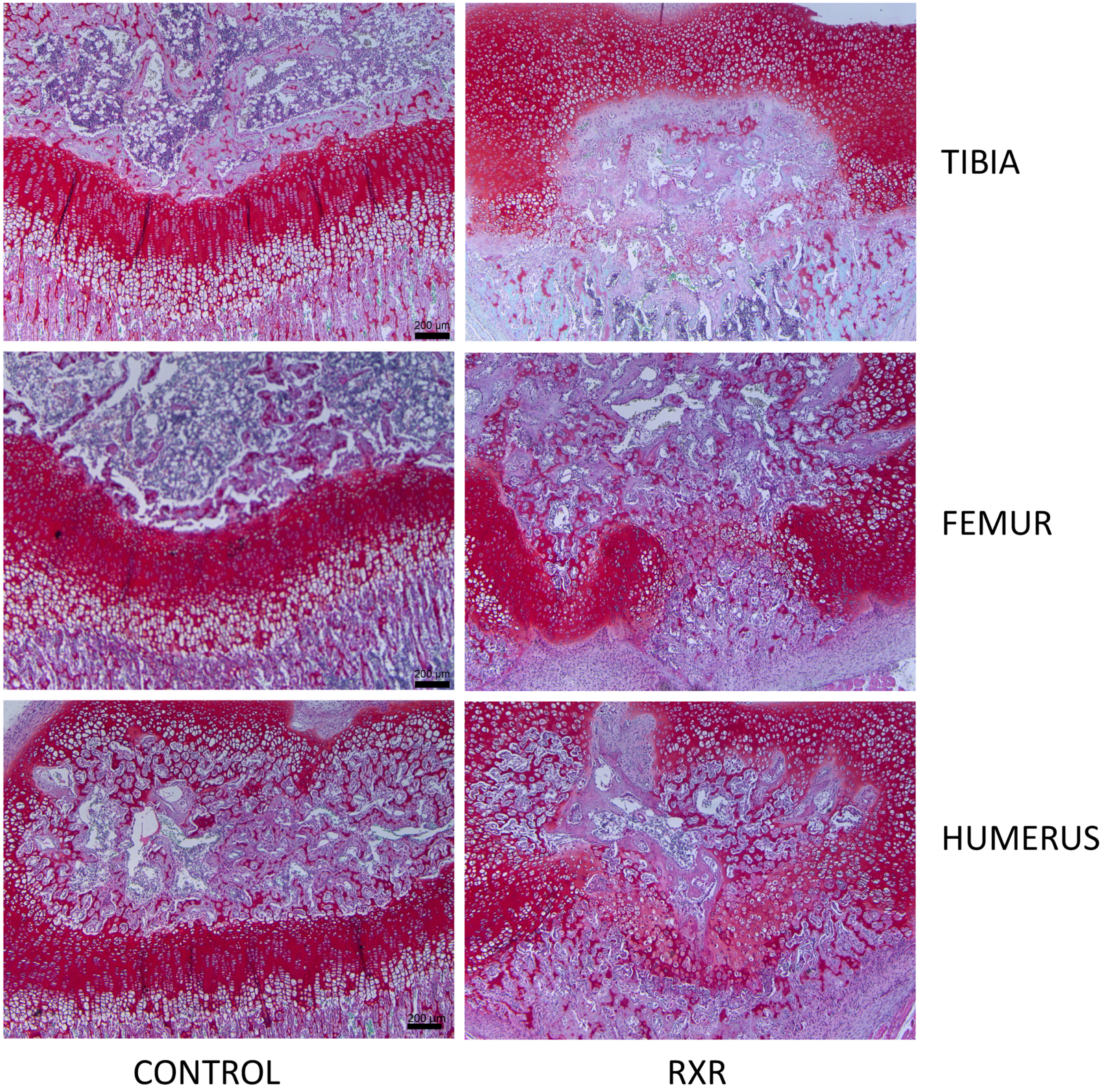

Suppl. Fig. 1: Disrupted Growth Plate Morphology in P16 Female Rat Long Bones. Safranin O / fast green staining of bone sections highlights appearance of disturbed growth plate organization and fusion of primary and secondary ossification centers in the RXR agonist-treated females (scale bar = 200µm).
